# Imaging spectrum of renal oncocytomas: a pictorial review with pathologic correlation

**DOI:** 10.1007/s13244-014-0373-x

**Published:** 2014-12-14

**Authors:** Kousei Ishigami, Aaron R. Jones, Laila Dahmoush, Leandro V. Leite, Marius G. Pakalniskis, Thomas J. Barloon

**Affiliations:** 1 Department of Radiology, University of Iowa Hospitals and Clinics, 3885 JPP, 200 Hawkins Drive, Iowa City, IA 52242 USA; 2 Department of Pathology, University of Iowa Hospitals and Clinics, Iowa City, IA USA

**Keywords:** Renal oncocytoma, Enhancement pattern, Radiological-pathological correlation, Diagnostic problems

## Abstract

**Objectives:**

The purpose of this pictorial review is to present the imaging spectrum of renal oncocytomas with radiological-pathological correlation.

**Conclusion:**

The differences in tumour cellularity (high cellularity or low cellularity with abundant stroma) and haemorrhagic/cystic change contribute to a wide spectrum of imaging findings of renal oncocytomas. Imaging findings substantially overlap those of common subtypes of clear cell and non-clear cell renal cell carcinomas. Multifocal renal oncocytomas are not rare, and making the diagnosis of oncocytoma with concomitant renal cell carcinoma is difficult. In addition, renal oncocytomas that demonstrate interval growth or develop in the setting of end-stage renal disease may be mistaken for malignancy.

***Teaching Points*:**

• *High cellular components demonstrate avid arterial enhancement and subsequent washout*.

• *Low cellular components demonstrate gradual subsequent enhancement owing to abundant stroma*.

• *Cystic and hemorrhagic changes may account for lesion heterogeneity in the delayed phase*.

• *Multifocal oncocytomas and oncocytomas coexisting with renal cell carcinoma are not rare*.

• *Renal oncocytomas may demonstrate interval growth*.

## Introduction

**Fig. 1:**
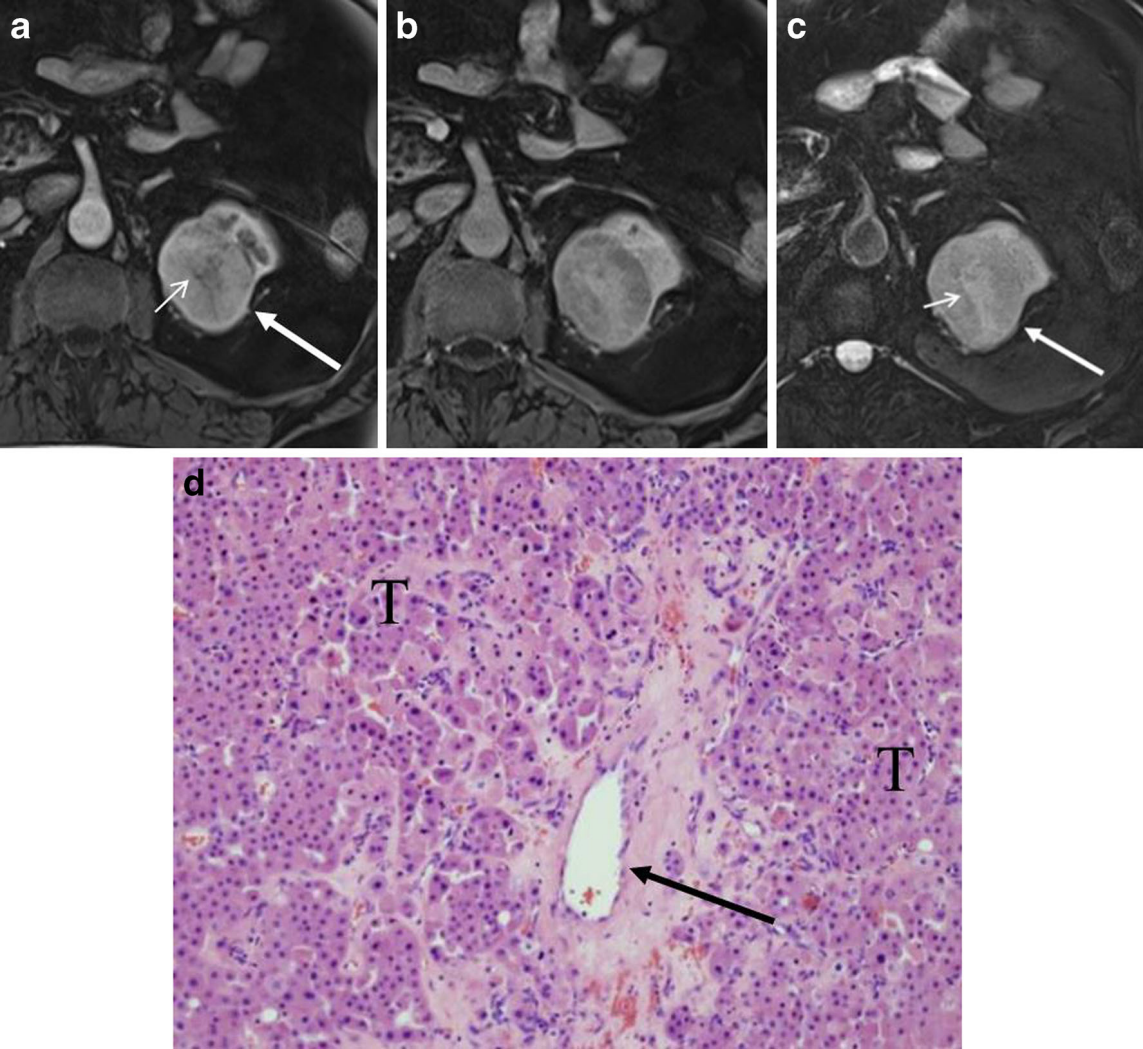
A 71-year-old man with classic renal oncocytoma in the left kidney. **a** The corticomedullary phase of the axial T1-weighted gradient echo (GRE) magnetic resonance (MR) image with fat saturation demonstrates an arterially enhancing mass (*arrow*) with hypointense central scar (*small arrow*). **b** The nephrographic phase demonstrates washout with enhancing central scar. **c** The axial T2-weighted fast spin echo (FSE) image with fat saturation shows the tumour to be of moderate signal (*arrow*) with hyperintense central scar (*small arrow*). **d** (High magnification) Oncocytoma, organoid pattern, tightly packed nests of tumour cells (*T*) with visible capillaries in the stroma (*arrow*)

Renal oncocytoma is a benign renal tumour, accounting for approximately 3–7 % of all renal tumours [1]. Typical imaging findings of renal oncocytoma are described as a homogeneous hypervascular mass with subsequent washout in the delayed phase [2, 3] (Fig. 1). A central scar is a characteristic finding, especially in a large oncocytoma [4]. However, such classic imaging findings are not common [5]. In many cases, renal oncocytomas are surgically resected because preoperative imaging diagnosis is not reliable to distinguish oncocytoma from renal cell carcinoma (RCC).

**Fig. 2:**
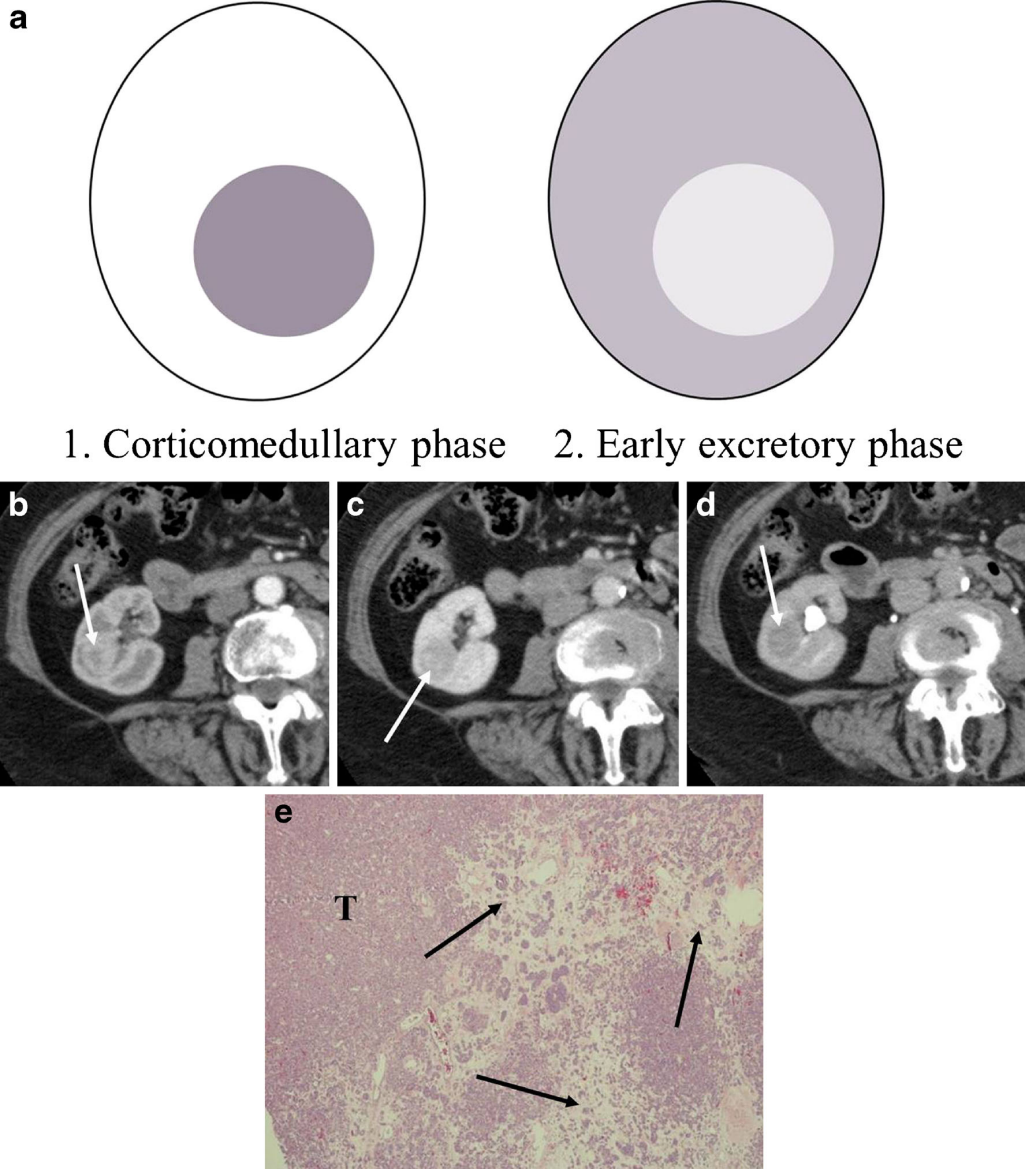
A schema of segmental enhancement inversion and a case of a 76-year-old woman with right renal oncocytoma demonstrating segmental enhancement inversion. **a** Schema of segmental enhancement inversion. *1.* A tumour shows two distinct areas of hyper-enhancement (*white*) and hypo-enhancement (*dark grey*) on the corticomedullary phase (30–40 s). Hyper- and hypo-enhancing areas consist of higher and lower cellularity components, respectively. *2.* On the early excretory phase (120–180 s), the higher cellularity component becomes relatively low attenuation due to contrast washout. The lower cellularity component becomes relatively high attenuation due to gradual enhancement of oedematous stroma. Therefore, contrast enhancement of these two distinct areas reverse between the corticomedullary (*1*) and the early excretory (*2*) phases. The peak enhancement of the oedematous stroma and the optimal timing of segmental enhancement inversion may be variable in each tumour. **b** The corticomedullary phase of the axial contrast-enhanced (CE) computed tomography (CT) demonstrates an intensely enhancing mass with a small central portion of low attenuation (*arrow*). **c** The low attenuation central portion in the nephrographic phase is relatively hyperattenuating in the corticomedullary phase (*arrow*); the relative enhancement is reversed. **d** The delayed phase shows the mass to be nearly homogeneous (*arrow*). **e** (Low magnification) Oncocytoma, in addition to the tightly packed nests of tumour cells (*T*), less densely arranged nests are located in an oedematous stroma (*arrows*)

Kim et al. [6] found that segmental enhancement inversion based on the corticomedullary and early excretory phase was a characteristic enhancement pattern of oncocytoma (Fig. 2). Segmental enhancement inversion is a term defined as a renal lesion that has two distinct zones of enhancement which show inverse patterns between the corticomedullary (30–40 s) and early excretory (120–180 s) phases. One zone is hyper-enhancing on the corticomedullary phase, which subsequently becomes hypo-enhancing on the early excretory phase. The other zone is hypo-enhancing on the corticomedullary phase and becomes hyper-enhancing on the early excretory phase [6, 7]. However, other studies have found it controversial whether or not segmental enhancement inversion is characteristic for oncocytoma [7–10]. Additionally, several studies examining the discrimination of RCC from oncocytoma based on the corticomedullary phase have shown inconsistent results [2, 11–13].

Therefore, renal oncocytomas demonstrate various imaging findings. Not only may the discrimination of oncocytoma from RCC be challenging, but also the presence of oncocytoma with concurrent RCC may be a diagnostic problem. Although imaging findings may not reliably discriminate oncocytoma from RCC, it is meaningful for radiologists to understand the wide imaging spectrum of oncocytomas when approaching the differential diagnosis of renal tumours.

The purpose of this pictorial review is to present the imaging spectrum of renal oncocytomas. To aid in understanding the imaging findings of oncocytomas, radiological-pathological correlations are provided.

## The diagnostic difficulties of discriminating renal oncocytoma from renal cell carcinoma

**Fig. 3:**
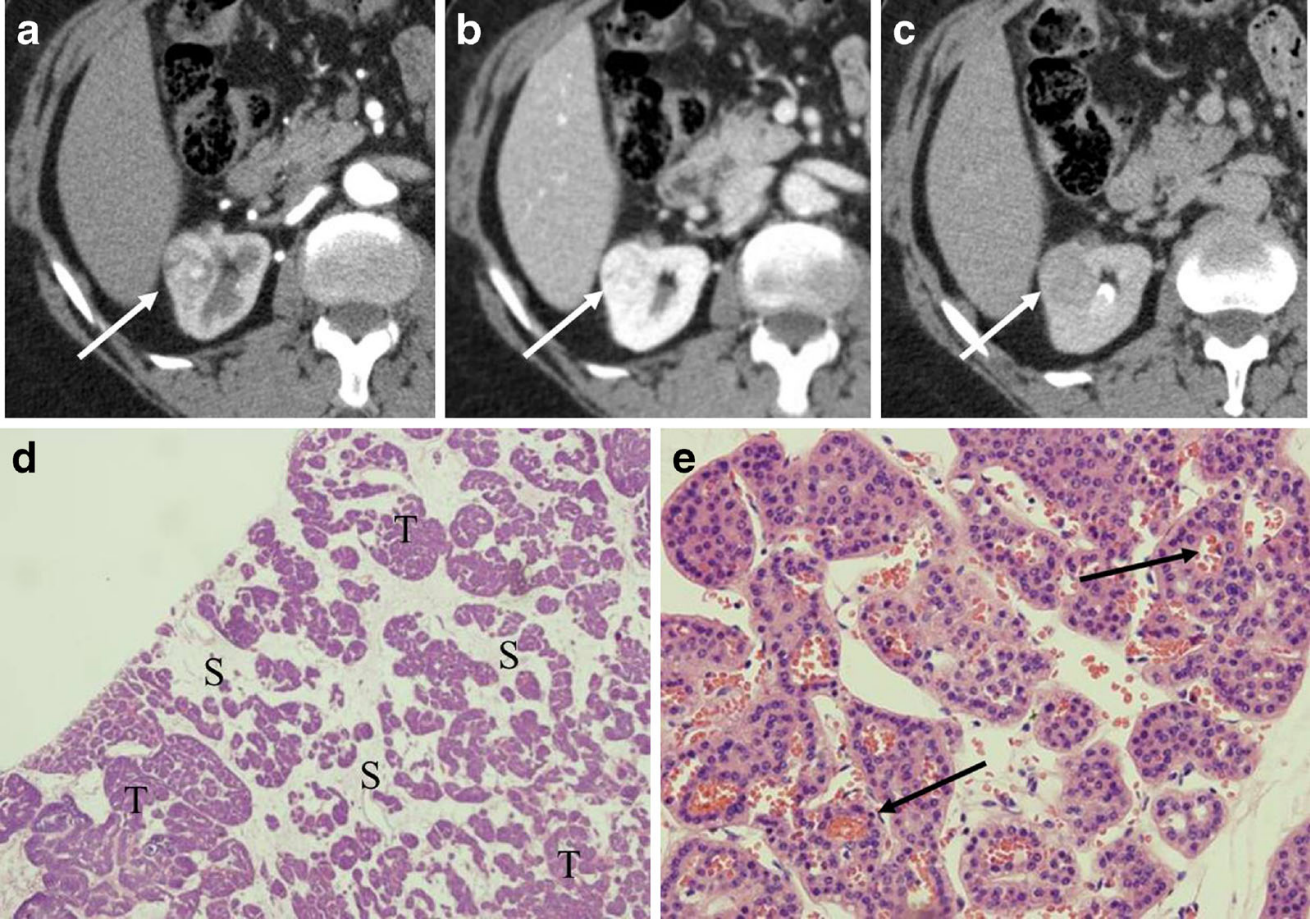
A 72-year-old woman with a right renal oncocytoma demonstrating persistent enhancement in the nephrographic phase. **a** The arterial phase of the axial CE-CT shows a heterogeneously enhancing mass (*arrow*). **b** The nephrographic phase shows avid enhancement of the mass (*arrow*). **c** The delayed phase shows the mass to washout to homogeneously low attenuation (*arrow*). **d** (Low magnification) Oncocytoma, equal amounts of tumour cells (*T*) and stoma (*S*). **e** (High magnification) Oncocytoma, organoid pattern with classic polygonal tumour cells with abundant granular eosinophilic cytoplasm, uniformly round nuclei with prominent nucleoli. Abundant small blood vessels are interposed within the stroma (*arrows*)

**Fig. 4:**
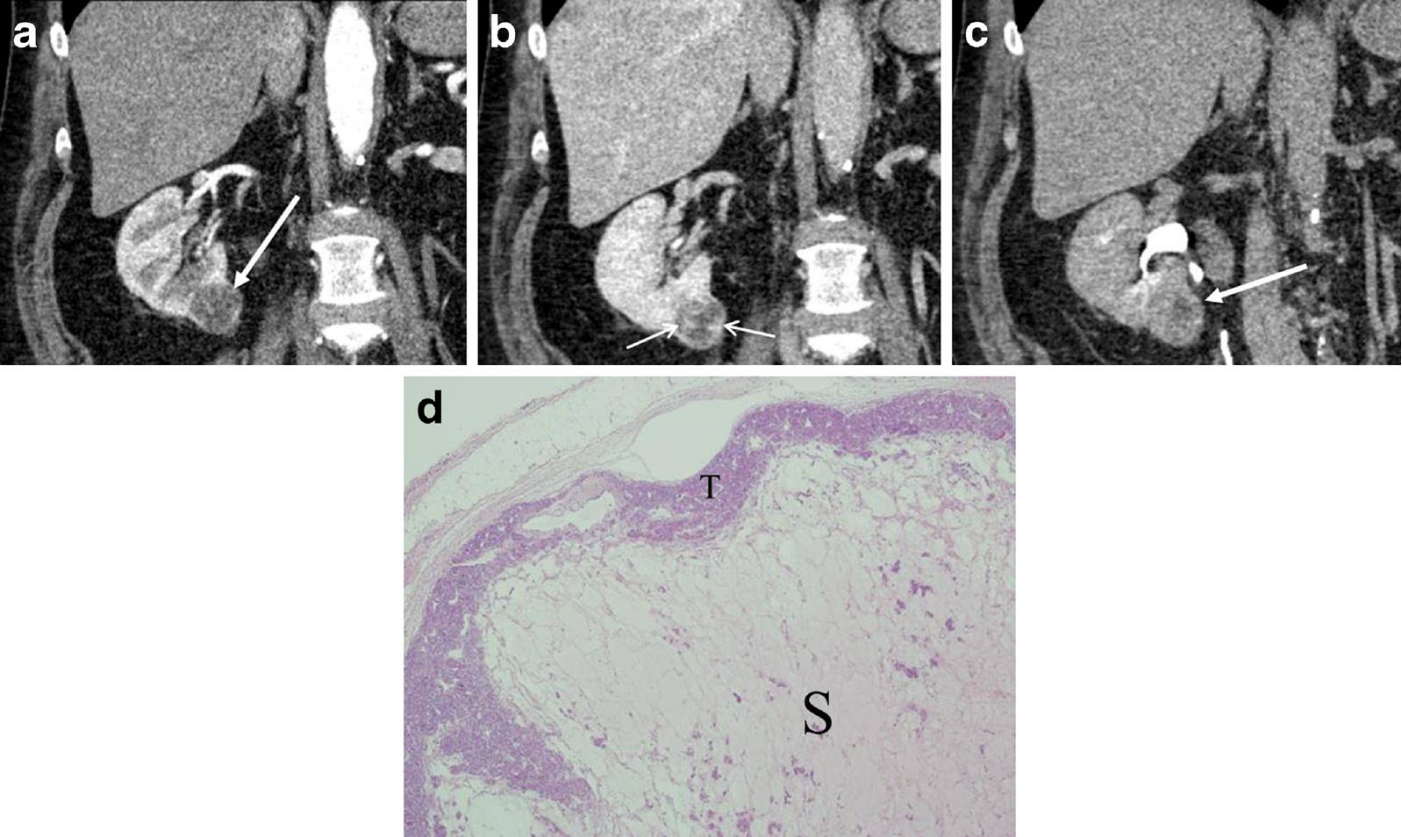
A 56-year-old man with right renal oncocytoma demonstrating hypovascularity and gradual enhancement. **a** Coronal reformatted image of the corticomedullary phase of a CE-CT shows a low attenuation mass in the lower right renal pole (*arrow*). **b** The nephrographic phase demonstrates patchy peripheral enhancement (*small arrows*). **c** The delayed phase shows gradual enhancement with a small residual area of low attenuation (*arrow*). **d** (Low magnification) Oncocytoma, a peripheral rim of tumour cells (*T*) surrounds abundant oedematous stroma (*S*)

**Fig. 5:**
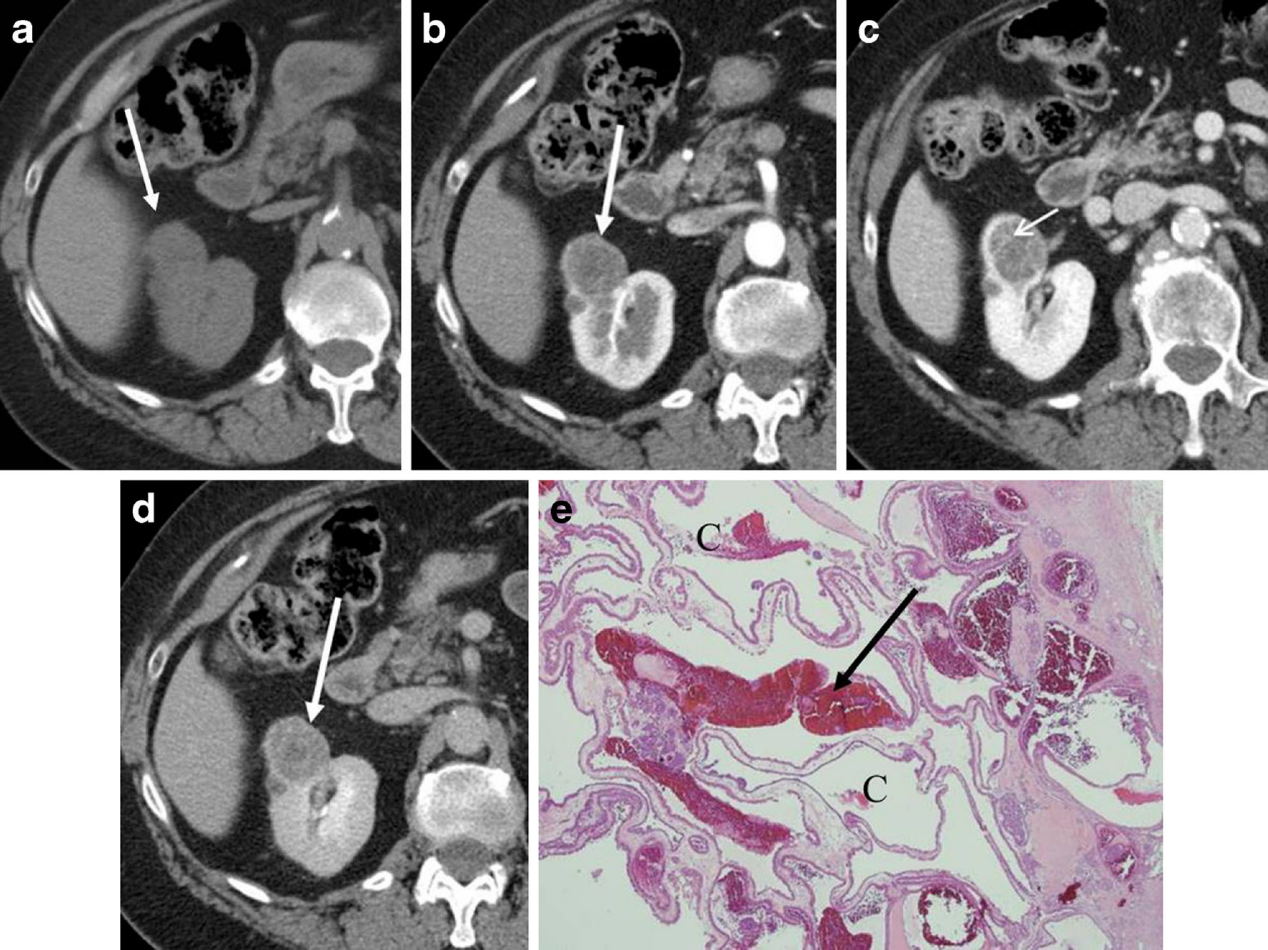
A 75-year-old woman with a right renal oncocytoma presenting as a hypovascular mass secondary to cystic change and haemorrhage. **a** Unenhanced axial CT image shows an exophytic right renal mass, which is iso-attenuating to the normal kidney (*arrow*). **b** The corticomedullary phase of the axial CE-CT shows the mass to be low attenuation (*arrow*). **c** The nephrographic phase demonstrates thin enhancing septations (*small arrow*). **d** The delayed phase shows the mass to be heterogeneous (*arrow*). **e** (Low magnification) Oncocytoma, cystic pattern, tumour cells predominantly arranged as cystic structures (*C*) with haemorrhage (*arrow*).

**Fig. 6:**
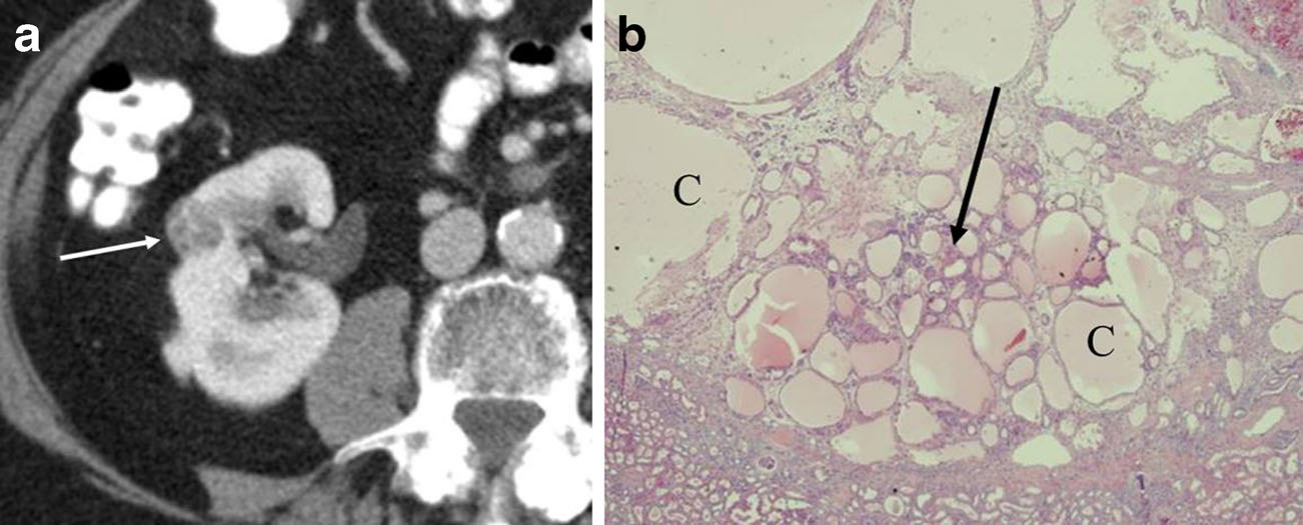
An 80-year-old woman with a right renal oncocytoma with cystic change. **a** The corticomedullary phase of the axial CE-CT demonstrates a small multilocular cystic right renal mass (*arrow*). **b** (Low magnification) Oncocytoma, tubulocystic pattern, tumour cells arranged as variably-sized cysts (*C*) and tubules (*arrow*)

Several studies examining the discrimination of clear cell RCC from oncocytoma based on arterial enhancement have revealed inconsistent results. For example, Gakis et al. [2] and Bird et al. [11] described that oncocytomas demonstrated greater enhancement than clear cell RCC in the corticomedullary phase. On the other hand, Young et al. [12] described that clear cell RCC demonstrated greater enhancement than oncocytoma. Pierorazio et al. [13] described that peak enhancement of clear cell RCC was seen predominantly in the corticomedullary phase, while that of oncocytoma was seen predominantly in the nephrographic phase (Fig. 3). Zhang et al. [14] described that oncocytoma commonly showed avid enhancement in the venous phase. Furthermore, oncocytoma may present as a hypovascular mass with gradual contrast enhancement (Fig. 4) or a persistently hypovascular or cystic mass (Figs. 5 and 6). Therefore, in oncocytoma, the degree of tumoural enhancement and the timing of peak enhancement are variable.

**Fig. 7:**
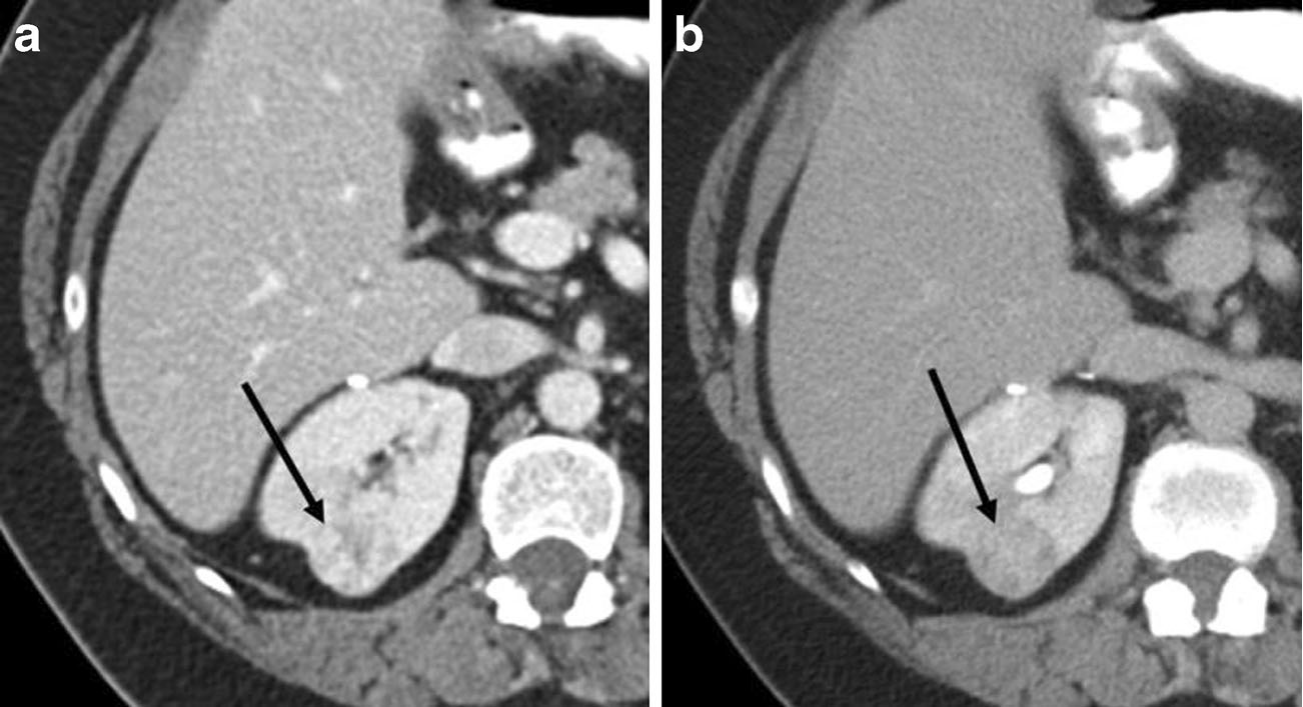
A 38-year-old woman with clear cell RCC of the right kidney mimicking segmental enhancement inversion (*see* Fig. 2). **a** The nephrographic phase of the axial CE-CT demonstrates avid contrast enhancement and an area of central low attenuation (*arrow*). **b** The enhancing area in the nephrographic phase (**a**) washes out to low attenuation in the delayed phase. The low attenuation area in the nephrographic phase shows relatively high attenuation (*arrow*)

Kim et al. [6] found that segmental enhancement inversion based on the corticomedullary and early excretory phases was a characteristic enhancement pattern of a relatively small (<4 cm) oncocytoma. In contrast, Millet et al. [8] did not find segmental enhancement inversion in oncocytoma or RCC, and O’Malley et al. [9] concluded that segmental enhancement inversion was not a common or characteristic finding for oncocytoma. Additionally, the enhancement pattern of RCC (clear cell RCC or chromophobe RCC) may mimic segmental enhancement inversion [15, 16] (Fig. 7).

**Fig. 8:**
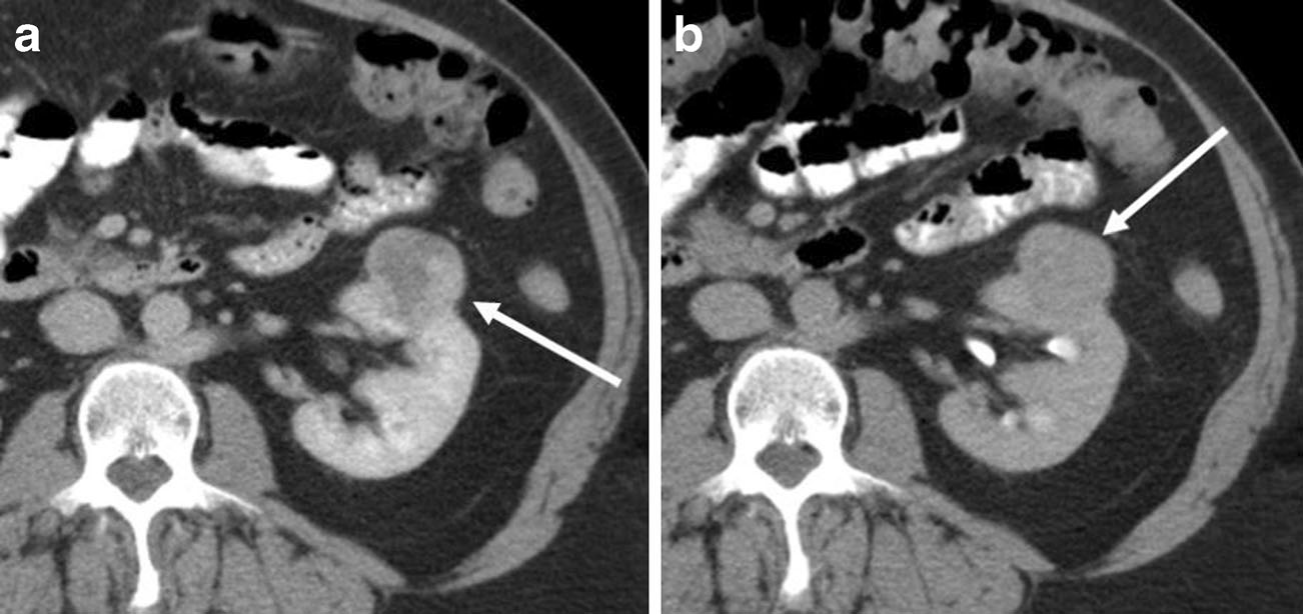
A 51-year-old woman with clear cell RCC of the left kidney, showing homogeneity in the delayed phase. **a** The nephrographic phase of the axial CE-CT demonstrates a left renal mass consisting of two distinct areas of relative hyper-enhancement (*arrow*) and hypo-enhancement. **b** In the delayed phase, the mass becomes homogeneous (*arrow*)

McGahan et al. [10] described that the most common feature of oncocytoma (<4 cm) was a heterogeneous enhancing mass that became homogeneous on the delayed phase (Figs. 2 and 3). Woo et al. [17] described that when segmental enhancement inversion was inconspicuous in the early excretory phase, oncocytomas might appear nearly homogeneous, especially in the case of small oncocytomas. However, similar finding may be seen in RCC (Fig. 8). Therefore, a characteristic enhancement pattern for renal oncocytomas has not been widely accepted.

**Fig. 9:**
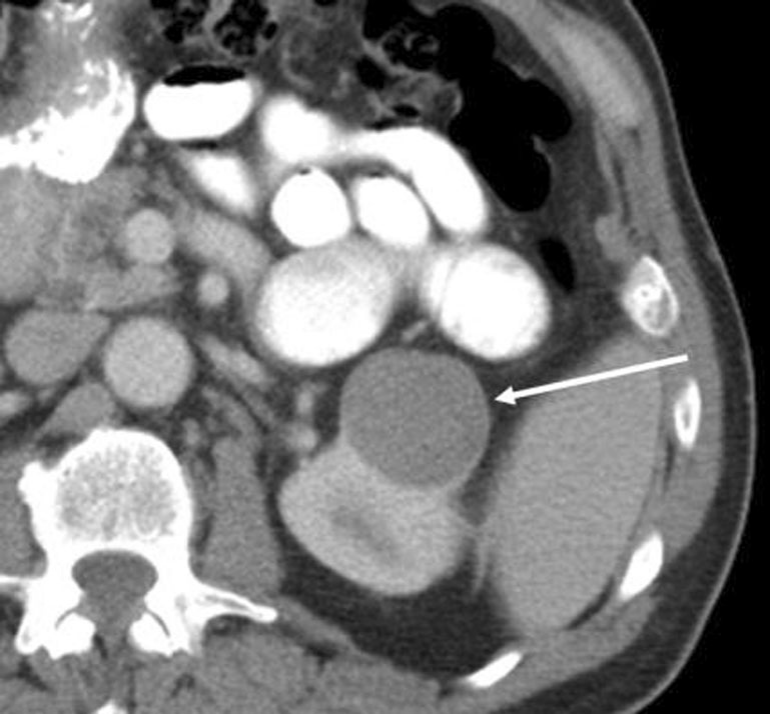
A 64-year-old man with papillary RCC of the left kidney. The nephrographic phase of the axial CE-CT demonstrates a homogeneously low attenuation mass (*arrow*)

Vascularity and enhancement patterns of oncocytomas are various, and oncocytomas can present as an either homogeneous or heterogeneous mass. Such a wide spectrum of imaging findings substantially overlaps those of common subtypes of RCCs. When an oncocytoma presents as a heterogeneous hypervascular mass, it may mimic clear cell RCC [14]. When an oncocytoma presents as a homogeneous hypovascular mass, it may mimic chromophobe or papillary RCCs on CT (Fig. 9).

## Radiological-pathological correlation of renal oncocytoma

Imaging findings and pathological correlations are summarised in Table 1.

**Table 1:** Imaging findings and pathological correlations

Imaging findings	Pathology findings
Intense arterial enhancement and subsequent washout	High cellularity (organoid pattern)
Segmental enhancement inversion	Two distinct areas of high cellularity and low cellularity (oedematous stroma) components
Arterial enhancement that persists in the nephrographic phase	Mixture of organoid pattern with rich vascularity and oedematous stroma
Hypovascularity and gradual enhancement	Abundant oedematous stroma (low cellularity)
Hypoehancement in the corticomedullary and delayed phase	Cystic and/or haemorrhagic change

There are three cellular patterns of renal oncocytomas, including organoid (Figs. 1d and 3e), tubulocystic and mixed pattern [4]. The organoid pattern is characterised by nests of tumour cells surrounded by a reticular framework of thin blood vessels and stroma [4]. The tumour nests can be tightly packed (high cellularity) or loosely arranged within an oedematous stroma (low cellularity) [4]. The tubulocystic pattern (Fig. 6b) is characterised by tumour cells arranged as tubular and cystic structures separated within an oedematous stroma (low cellularity) [4].

Abundant vascularity in the organoid pattern may account for the arterial enhancement of oncocytomas. In the organoid pattern, tightly packed tumour nests may explain washout patterns in the venous phase owing to its high cellularity. For example, if an oncocytoma predominantly consists of tightly packed tumour nests, the tumour would show avid arterial enhancement and subsequent washout in the nephrographic phase (Fig. 1). On the other hand, the presence of abundant stroma (low cellularity) may explain slow or gradual enhancement. If an oncocytoma contains abundant stroma, the tumour would present as a hypovascular mass or gradually enhancing lesion (Fig. 4). If an oncocytoma consists of two distinct components of high and low cellularity, the tumour may demonstrate segmental enhancement inversion (Fig. 2). In fact, Kim et al. [6] described that early-enhancing and delayed-enhancing components in oncocytomas (segmental enhancement inversion) reflected areas of compactly arranged tumour cells (high cellularity) and abundant stroma, respectively.

The difference in the cellularity (and stroma) may influence the degree of tumour enhancement and characteristic enhancement pattern of individual oncocytomas.

**Fig. 10:**
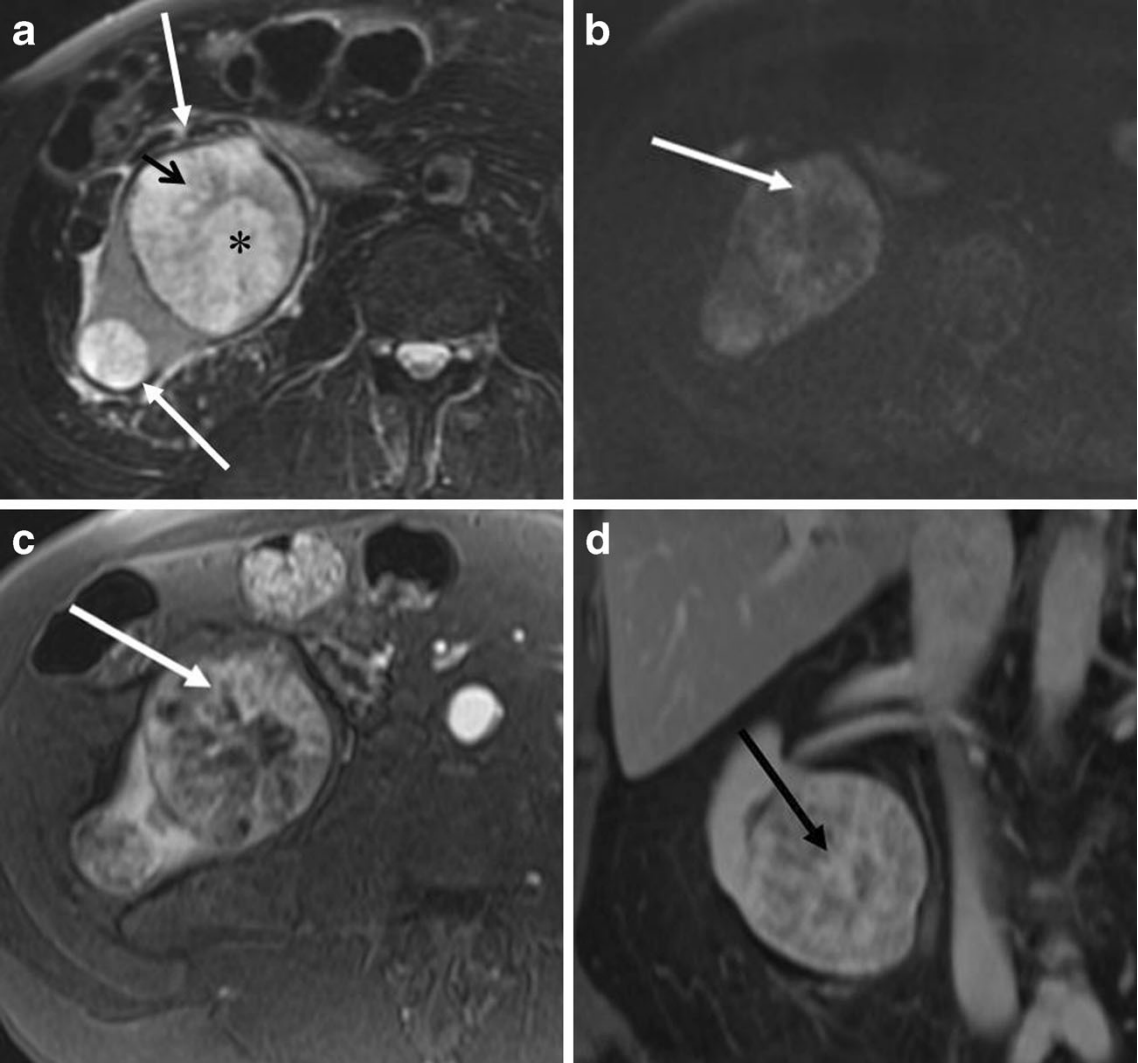
A 74-year-old woman with multifocal renal oncocytomas. **a** The axial T2-weighted FSE MR image with fat saturation shows two heterogeneous masses in the right kidney (*arrows*). The tumours consist of a mixture of intermediate (*small arrow*) and high signal (*asterisk*) components. **b** The axial diffusion weighted image (*b* factor = 800) shows the area of moderate signal on the T2-weighted image to be relatively high signal (*arrow*), suggesting increased cellularity. **c** The arterial phase of the axial T1-weighted GRE image with fat saturation demonstrates the area of intermediate signal on the T2-weighted image to be hypervascular (*arrow*). **d** Post-contrast coronal T1-weighted GRE image with fat saturation shows partial enhancement in the area of increased signal on the T2-weighted image (*arrow*)

**Fig. 11:**
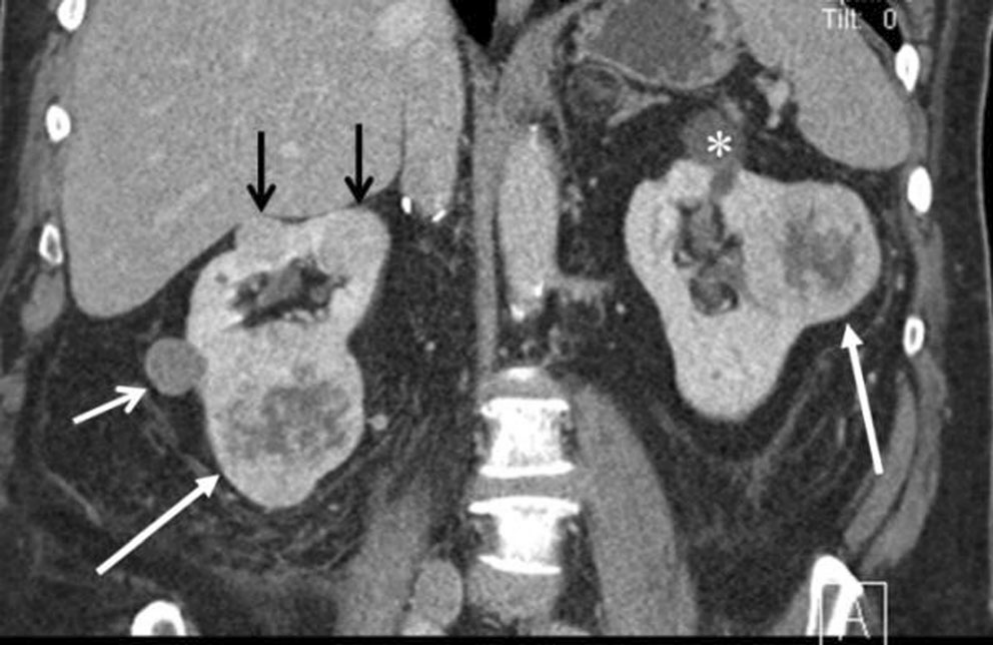
A 64-year-old man with bilateral multifocal oncocytomas coexisting with papillary RCC of the right kidney. The coronal reformatted image in the nephrographic phase of the CE-CT demonstrates five solid masses (*arrows*) and one simple cyst (*asterisk*). An exophytic right renal mass with relatively decreased contrast enhancement (*smaller white arrow*) was found to be papillary RCC. The four remaining enhancing masses were oncocytomas. Note that the small tumours are homogeneous (*smaller black arrows*), while the larger tumours are relatively heterogeneous (*larger white arrows*)

The prevalence of cystic change and haemorrhage in renal oncocytomas ranges from 5 % to 20 % [1, 18, 19]. Haemorrhage and cystic change may alter the imaging findings of oncocytomas to present as a heterogeneous mass, especially in a larger tumour [17] (Figs. 10 and 11). In addition, partial haemorrhage and cystic change may account for lesion heterogeneity in the delayed phase, demonstrating a focal non-enhancing area within the tumour.

When cystic changes and haemorrhage are extensive, oncocytomas may present as a hypovascular mass (Fig. 5). Oncocytomas may present as a multilocular cystic mass [20] (Fig. 6). In addition, a rare variant of telangiectatic oncocytoma has been reported, which is histologically characterised by a predominance of multicystic spaces filled with blood products [21].

## Magnetic resonance imaging findings

On magnetic resonance imaging (MRI), the high cellular component demonstrates moderate signal on T2-weighted images and relatively high signal on diffusion-weighted imaging (Figs. 1 and 10). High T2 signal within the tumour may reflect oedematous stroma, central scar or cystic change. Dynamic contrast-enhanced MRI findings are similar to CT; the components of high cellularity demonstrate arterial enhancement and the component of high T2 signal shows gradual enhancement with partial or complete fill-in [3] (Fig. 10). It has been reported that apparent diffusion coefficients (ADCs) of oncocytomas were significantly higher than that of RCC [22]. However, it is questionable whether diffusion-weighted imaging is useful in the differential diagnosis of oncocytoma versus RCC due to the variability of oncocytoma cellularity.

## Other uncommon imaging manifestations

### Calcification

**Fig. 12:**
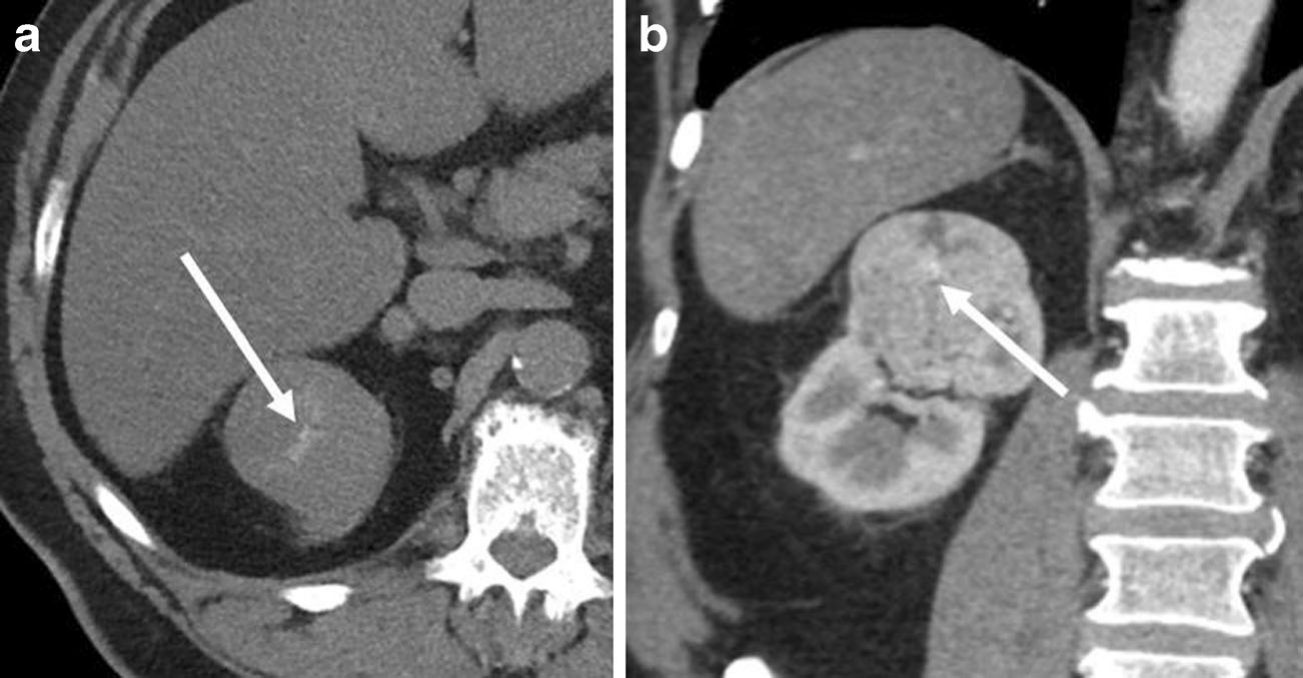
A 71-year-old man with a right renal oncocytoma with calcification. **a** The axial unenhanced CT demonstrates calcification (*arrow*) within the mass. **b** The coronal reformatted image in the corticomedullary phase shows calcification in the central scar (*arrow*)

Although the pathological literature describes calcification in 31 % of oncocytomas [1], calcification is relatively uncommon on CT. Calcification is typically present within the central scar [1, 5] (Fig. 12).

### Multifocal oncocytoma

**Fig. 13:**
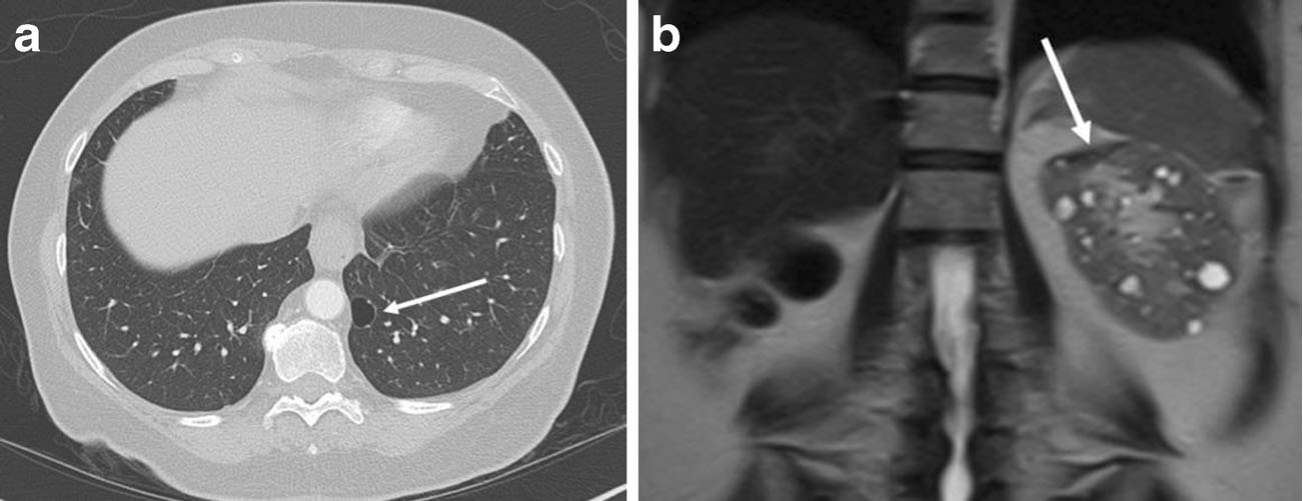
A 65-year-old woman with Birt-Hogg-Dube syndrome (previous history of RCC and oncocytomas). **a** The axial chest CT shows a pulmonary cyst in the left lung base (*arrow*). **b** The coronal single shot FSE T2-weighted image demonstrates multiple renal cysts. The *arrow* depicts post-surgical change of previous partial nephrectomy

Oncocytomas are multifocal in 2.5–16 % of cases and bilateral in 4–12 % [23]. Multifocal oncocytomas (Figs. 10 and 11) can be either sporadic or associated with Birt-Hogg-Dube syndrome. Birt-Hogg-Dube syndrome is a rare autosomal dominant disease that is characterised by cutaneous hair follicle tumours and multiple renal tumours including RCC and oncocytoma. Renal and pulmonary cysts are also associated with the syndrome [24] (Fig. 13). The presence of pulmonary cysts helps to discriminate Birt-Hogg-Dube syndrome from von-Hippel Lindau disease [24]. Renal oncocytosis (multiple oncocytic lesions) is a recently established disease entity defined as diffuse replacement of the renal parenchyma by numerous oncocytic tumours, such as hybrid tumours, chromophobe RCCs, renal oncocytomas, and oncocytic renal parenchyma [25]. Renal oncocytosis may occur sporadically or may be associated with chronic renal failure and long-term haemodialysis [25].

### Oncocytoma coexisting with renal cell carcinoma

**Fig. 14:**
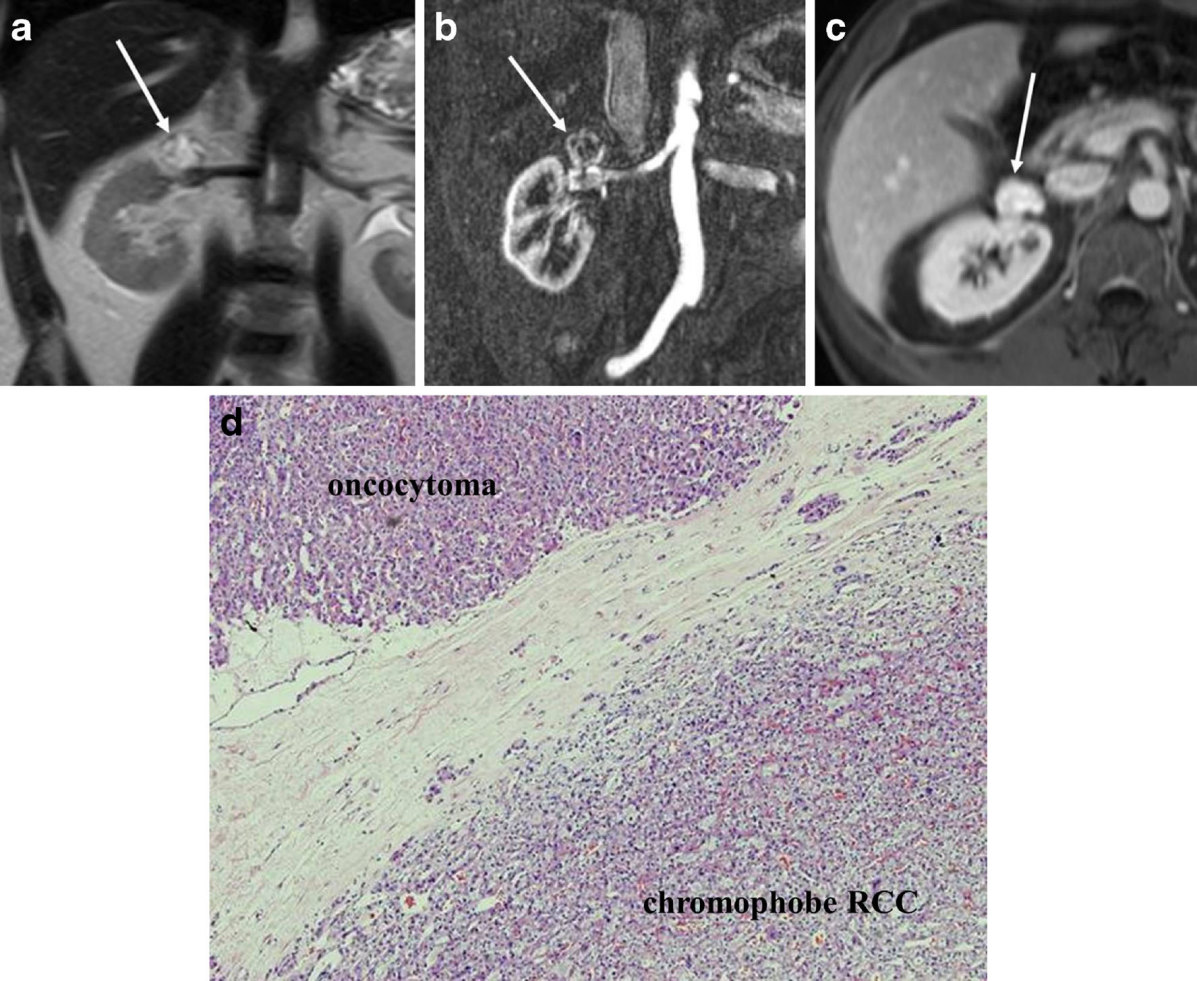
A 54-year-old man with a hybrid tumour (oncocytoma containing chromophobe RCC component) in the right kidney. **a** The coronal T2-weighted single shot FSE MR image shows a right upper renal pole mass with mixed high and intermediate signal intensity (*arrow*). **b** The corticomedullary phase of the coronal T1-weighted GRE image shows heterogeneous enhancement of the mass. **c** The axial post-contrast T1-weighted GRE image with fat saturation shows persistent and homogenous enhancement of the mass (*arrow*). This oncocytoma also demonstrates a lobular morphology. **d** (Low magnification) Hybrid tumour: *upper left* oncocytoma; *lower right* eosinophilic variant of chromophobe renal cell carcinoma (chromophobe RCC)

Coexisting RCC is not rare in patients with oncocytoma, and the reported incidence is up to 10 % [26]. Coexisting RCC may present as an incidental microscopic finding sometimes associated with oncocytoma (hybrid tumour) (Fig. 14) or as a separate mass in the ipsilateral or contralateral kidney [27] (Fig. 11).

Renal oncocytomas and chromophobe RCC may be related entities (oncocytic lesions). These two tumours may arise from a common precursor with the potential to differentiate into either a benign (oncocytoma) or malignant (chromophobe RCC) lesion [23, 24]. Hybrid tumours consisting of oncocytoma and chromophobe RCC components have been described [25, 28]. The lesion heterogeneity on imaging does not indicate the presence of chromophobe RCC and imaging findings are not helpful for making the diagnosis of a hybrid tumour (Fig. 14). In addition, even though metastatic oncocytomas have been reported, they may potentially be chromophobe RCCs misdiagnosed as oncocytomas [4, 29, 30].

## Findings that may be mistaken for malignancy

### Interval growth

**Fig. 15:**
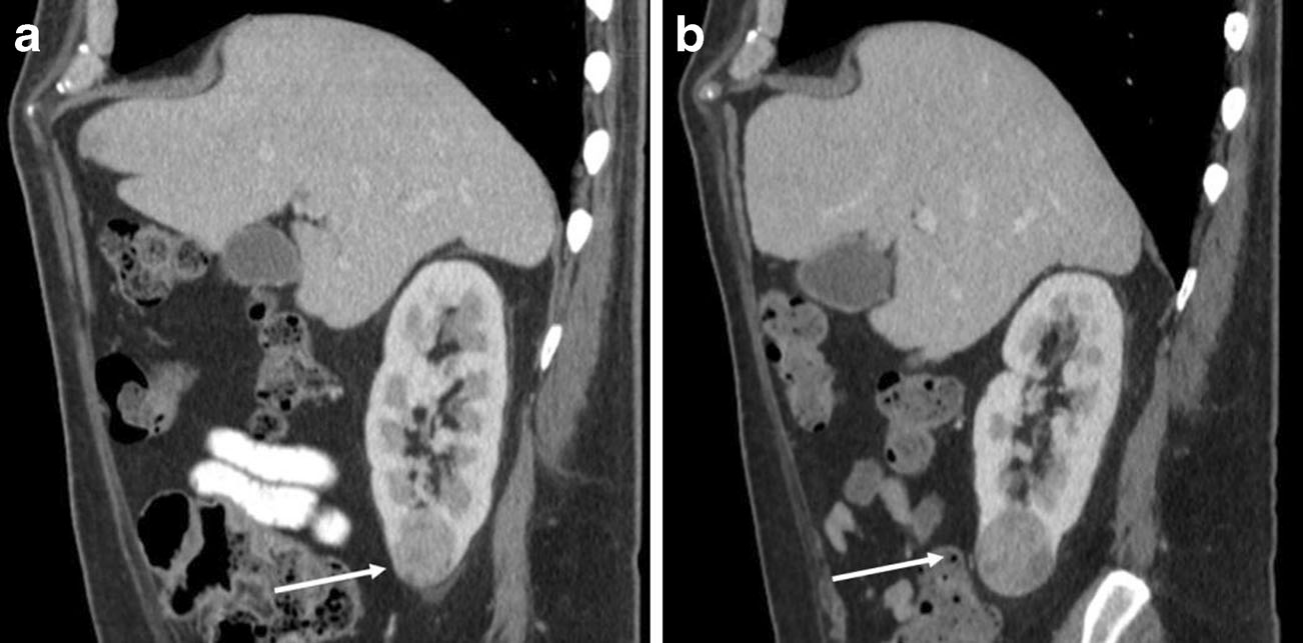
A 61-year-old man with a right renal oncocytoma demonstrating interval growth. **a** The sagittal reformatted image in the portal venous phase of a CE-CT demonstrates a moderately enhancing well-circumscribed mass in the lower right renal pole (*arrow*). The mass measures 3.5 cm in long axis diameter. **b** Follow-up CT 2 years after **a** demonstrates interval increased size of the mass (*arrow*), now measuring 4 cm

Oncocytomas may demonstrate slow interval growth (Fig. 15). Slight interval growth does not indicate malignancy and the growth rate is not helpful in discriminating RCC from oncocytoma because the growth rate of RCC is variable. The reported mean growth rate of clear cell RCC (0.7 cm/year) is not significantly different from that of oncocytomas (0.5 cm/year) [31].

### Extension to the perinephric fat

**Fig. 16:**
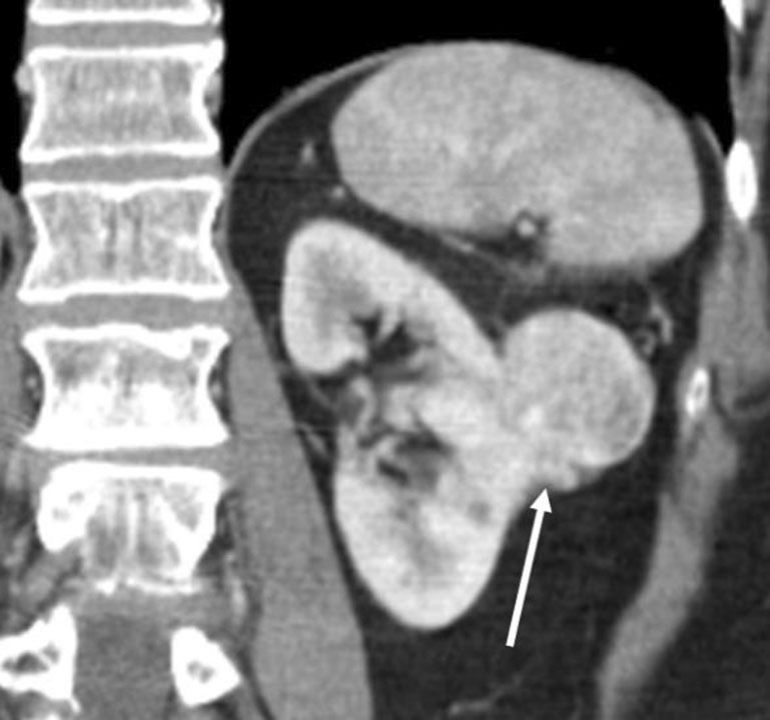
A 75-year-old woman with a left renal oncocytoma with perinephric fat extension. The coronal reformatted image of the portal venous phase of CE-CT shows tumour extension into the perinephric fat at the inferior aspect of the tumour (*arrow*)

The extension to the perinephric fat is considered atypical for oncocytoma [4, 29] (Fig. 16) and the clinical significance of this imaging finding is uncertain. While radiologically evident infiltrative growth indicates malignancy, oncocytomas typically present as a well-circumscribed or lobulated mass and associated perinephric fat extension is typically radiologically subtle (Fig. 16).

### Oncocytoma arising in end-stage renal disease

**Fig. 17:**
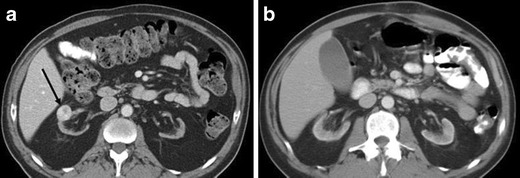
A 58-year-old man with a right renal oncocytoma arising from end-stage renal disease. **a** The portal venous phase of the axial CE-CT depicts a well-circumscribed enhancing mass (*arrow*) in the mid portion of the right kidney. Note that the bilateral kidneys are atrophic. **b** CE-CT 9 years prior demonstrates no evidence of a renal mass

Oncocytomas rarely develop in patients on haemodialysis [32] (Fig. 17). However, oncocytosis (multiple oncocytic lesions) may be associated with chronic renal failure and long-standing haemodialysis [33]. Because the pre-operative diagnosis of oncocytoma is difficult and the association between renal cell carcinoma and long standing dialysis is well-known, surgical treatment is warranted if the tumour is resectable [32].

## Conclusions

The wide spectrum of imaging findings regarding renal oncocytomas may be explained by the differences in the variable mixture of high-cellularity and low-cellularity components. High cellularity may explain intense arterial enhancement and subsequent washout. Low cellularity with abundant stroma may explain a lesser degree of arterial enhancement and gradual subsequent enhancement. Cystic change and haemorrhage may also contribute to lesion heterogeneity and the lesser degree of arterial enhancement.

The presence of segmental enhancement inversion may be suggestive of renal oncocytoma, although it can be seen in RCC. Oncocytomas tend to become homogeneous in the delayed phase. However, it may be more practical to consider that a heterogeneous mass in the delayed phase is more likely RCC because it is more common than oncocytoma. When the imaging findings are suspicious for oncocytoma, it is still necessary to perform biopsy because imaging findings of RCC and oncocytoma overlap. Additionally, biopsy may mischaracterise an RCC as an oncocytoma, and some oncocytomas may contain RCC components (hybrid tumour). Therefore, even though a biopsy specimen is suggestive of oncocytoma, careful imaging follow-up or less invasive therapeutic options such as nephron-sparing surgery, cryoablation or radiofrequency ablation may be indicated.
